# Zymolytic Grain Extract (ZGE) Significantly Extends the Lifespan and Enhances the Environmental Stress Resistance of *Caenorhabditis elegans*

**DOI:** 10.3390/ijms20143489

**Published:** 2019-07-16

**Authors:** Lu Hou, Mengying Jiang, Qiong Guo, Wei Shi

**Affiliations:** 1 School of Life Science, Jilin University, Changchun 130012, China; 2 Key Laboratory for Molecular Enzymology and Engineering of Ministry of Education, Jilin University, Changchun 130012, China

**Keywords:** zymolytic grain extract, *C. elegans*, lifespan elongation, ultraviolet radiation resistance, FUDR resistance

## Abstract

Many reports have shown that grains play an important role in our daily lives and can provide energy and nutrients to protect us from various diseases, and they are considered to be indispensable parts of our lives. It has been reported that some constituents in grains could exert functional effects against HIV infections and multiple cancers. Zymolytic grain can produce some new useful molecules and thus support the cell nutrients in the human body. In this study, the effects of zymolytic grain extract (ZGE) supernatants on the changes of nematode indicators were investigated, including lifespan, self-brood size, and body length in environmental conditions (temperature, ultraviolet radiation or 5-fluoro-2′-deoxyuridine (FUDR) stimuli). We found that, compared to the control group, the ZGE supernatant-feeding group could prolong the lifespan of nematodes under normal conditions. More importantly, ZGE supernatants could improve the ability of nematodes to resist stress. When the concentration of FUDR was 400 or 50 μM, the ZGE supernatant-feeding group could prolong lifespan by an average of 38.4% compared to the control group, and the eggs of the ZGE supernatant-feeding group could hatch and develop into adults. These results indicated that ZGE could protect *C. elegans* from external stress and thus prolong their lifespan and improve the physiological state of nematodes. Therefore, ZGE supernatant has potential to be used as a nutritional product in antioxidant and anti-aging research.

## 1. Introduction

Aging is the process of degenerative changes of organ tissues and functions [1]. Aging can reduce the body’s ability to maintain homeostasis under environmental stress, thereby increasing the incidence of illness and death. Previous reports have shown that aging is highly associated with hypertension, type 2 diabetes, atherosclerosis, Alzheimer’s disease [2] and other diseases [3]. There are many factors which could induce aging, such as environmental pollution, mental stress, heredity and others inside or outside of the body, as it relates to the reduction of regenerative cells, the weakness of organs, and the increasing of free radicals in the body. Extensive research has demonstrated that reactive oxygen species (ROS) play an important role in aging [4], and thus maintaining ROS at an appropriate level is important to keep balance in organisms [5]. With the increasing excess ROS level, oxidative stress could destroy the cell components, including by DNA damage, protein oxidation and lipid degradation [6], and trigger disease-related aging [7]. Recently, Federico Sesti et al. found that the up-regulation of K+ channel activity could reduce the neuronal excitability in a *Caenorhabditis elegans* model, which is one of the signs of brain aging [8]. Meanwhile, excessive ROS was present in the aging brain, and thus the oxidative modification of the K+ channel was shown to be probably one of the mechanisms of aging [8]. At the same time, ROS also plays an important role in the occurrence and development of various diseases. Hiroyuki Takano et al. found that ROS could cause the deterioration of cardiac function and participate in atherosclerosis, myocardial ischemia/reperfusion injury and heart failure [9], as well as the pathogenesis of inflammatory diseases [10] and meningitis [11] associated with periodontitis. These effects represent an imbalance between the production of ROS and the ability to repair damage in vivo [12]. In order to eliminate the impact of oxidative damage in vivo and reduce the harmful effects of peroxide damage on organisms, a series of antioxidants prepared in chemical synthesis are currently widely used such as butyl hydroxy anisol (BHA) [13], dibutyl hydroxy toluene (BHT) [14], propyl gallate (PG) [15], and sodium D-isoascorbate. However, in recent years, researchers have found that the use of synthetic antioxidants should be limited because of their potential health risks, such as the damage to the liver and the possibility of inducing cancers. Thus, it is necessary to develop an antioxidant that has a strong antioxidative capacity, few side effects and nutritional benefits. At present, many studies on the antioxidant activity of natural products have been reported. For example, Yang et al. showed that Ginseng and ginsenosides can promote DNA, RNA and protein synthesis to repair damaged DNA molecules or prevent their cross-linking, thereby decreasing or even reversing the aging process [16]. *Ganoderma lucidum* polysaccharide D6 has also been reported to promote protein synthesis in liver and bone marrow cells [17]. Similarly, Ding et al. identified that polysaccharides from *Tricholoma Lobayense* also had favorable antioxidative effects [18]. Therefore, it is of great importance to develop natural products with antioxidative and anti-aging properties.

Grains have been essential to human beings since ancient times. With the improvement of people’s living conditions, they not only provide nutrients as food but are also of benefit for health, and thus whole-grain food has become an option for health care. They also have the potential to improve weight management [19], ameliorate metabolic syndrome [20], reduce the risk of asthma, improve carotid health [21], and reduce inflammation [22] and the incidence of bowel cancer [23]. Also, the grains have been demonstrated to possess antioxidative properties [24]. The occurrence of these diseases is closely related to our daily living habits, including the refined grains in daily consumption and the lack of fibrous substance grains, micronutrients and phytochemicals which are contained in the brans [25] and the germ fractions of whole cereals [26], and the imbalance of energy intake. The reason why cereals can exert the above-mentioned positive physiological effects is inseparable from the complex chemical substances and elements contained in them. The phytochemicals contained in cereals, including fibers, oligosaccharides, fatty acids, lignin and polyphenols, have multiple physiological functions and recognized health benefits [27]. In particular, the antioxidative properties of polyphenols are also found to be positively correlated with antioxidant properties in plant seeds [28], leaves [29], grain extracts and fruits. In order to take advantage of the beneficial effects of different grains, a mixture of nine ingredients was processed by zymolysis with a series of enzymes to produce a zymolytic grain extract (ZGE). Previous research has found that ZGE could reduce gastrointestinal infections and assist in the treatment of dyspepsia and patients with impaired immunity. Besides this, it possessed suppressive efficacy for HIV-1 infections [30].

It has only been a few decades since a new era in aging research was inaugurated following the isolation of the first long-lived strains of *Caenorhabditis elegans*. *C. elegans* has the advantage of being the first model organism in which the genetic basis of aging was recognized [31]. *C. elegans* is useful for studying different aspects of aging because of its small size, rapid generation time (3 days at 20 °C) [32], ease of cultivation in inexpensive laboratory media and short adult lifespan (2 weeks at 20 °C). It responded to changes in environmental conditions such as temperature, oxygen content and noxious stimuli in easily assayable ways [33]. In addition, worms offer some unique advantages for aging research. Due to an invariant lineage, every adult worm has precisely 959 cells that make up its somatic tissues [34]. These can be easily examined because of their transparent bodies. Additionally, the genomic sequence of *C. elegans* is 40% homologous to humans [35], and some important signaling pathways are also highly conserved. Many human genetic diseases, such as muscle atrophy and Parkinson’s disease, are currently studied using the nematode model. Therefore, nematodes are a classic model in antioxidant research and can provide a good basis for the development of anti-aging drugs.

In this study, we aimed to investigate the antioxidant properties of ZGE using the model of *C. elegans* and to explore antioxidant bioactivity by testing the indexes of nematodes in terms of their lifespan, egg-laying rate and other aging-related behaviors.

## 2. Results

### 2.1. ZGE Does Not Inhibit the Propagation of E. coli OP50

**Figure 1:**
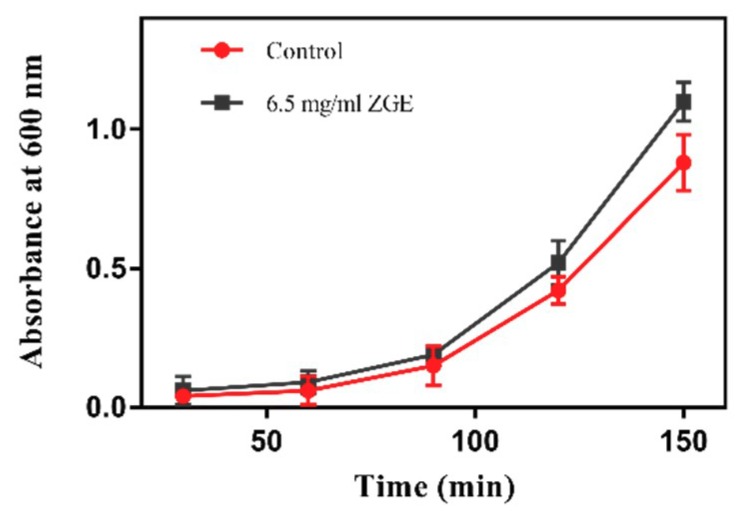
Effect of zymolytic grain extract (ZGE) on the propagation of *E.coli* OP50.

The metabolism and aging is inseparable. Compared with casual food, dietary restrictions can be consistently recognized to prolong life cycles and slow down the occurrence of various diseases caused by aging [36]. One of the most important factors in extending the life mechanism of nematodes is energy limitation, which limits the diet of nematodes. If biological cell nutrients have a strong effect on the inhibition of the *E. coli* OP50, the reason for their extended lifespan is probably caused by the nematode’s food being reduced for the group containing the biofilm in comparison to that of the control group, resulting in an energy limitation. As shown in Figure 1, compared with the control group, the growth rate of the OD_600_ value of ZGE group was faster, indicating that ZGE could promote the growth of OP50. Thus, we can conclude that the increasing nematode life cycle by the fermentation of the cereal nutrient solution was not caused by the energy constraints owing to insufficient nematode food. Meanwhile, it was indicated that ZGE changed the physiological indicators of nematodes not by changing the basic living conditions but through using its own biological activity to execute its functions. Therefore, we next tested a series of indicators to identify how ZGE influenced the cell cycle of nematodes.

### 2.2. ZGE Can Extend the Lifespan of C. Elegans N2 under Normal Culture Conditions

**Figure 2:**
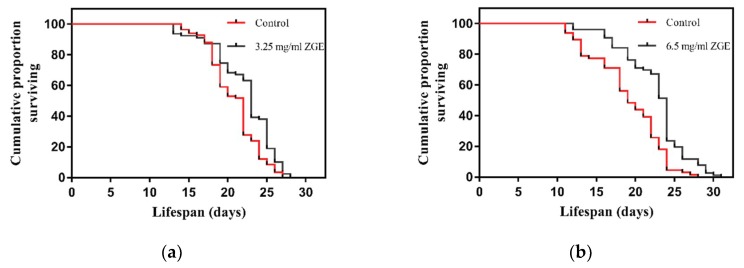
Life span analysis of nematodes in the presence (black) or absence (red) of ZGE. Compared with the control group (*N* = 68), ZGE at concentrations of 6.5 mg/mL (*N* = 73) (**a**) and 3.25 mg/mL (N = 73) (**b**) significantly extended the mean lifespan of wild-type *C. elegans* N2 by 13.6% and 7.6%, respectively. The data were processed using the Kaplan–Meier survival analysis tool in GraphPad Prism. *p* < 0.0001 (log-rank test).

To determine the life extension properties of ZGE, a lifespan assay was performed at 20 °C, which is an optimal temperature for wild-type *C. elegans* N2. Here, we found that ZGE had a significant ability to extend the lifespan of N2 at both concentrations tested (3.25 mg/mL and 6.5 mg/mL), as shown in Figure 2. Compared with the controls, ZGE increased the mean lifespan of the wild-type worms in a dose-dependent manner (7.6% at 3.25 mg/mL ZGE (a), 13.67% at 6.5 mg/mL ZGE (b)). Statistical analysis revealed that the maximum lifespan of the worms was increased to 28 days and 31 days by 3.25 mg/mL and 6.5 mg/mL ZGE, respectively. Thus, ZGE was demonstrated to exhibit a significant lifespan-extending effect in *C. elegans*. To further explore the influence of ZGE on life cycles, the nematodes were treated under abnormal conditions to analyze the effects of heat-stress and radiation stress.

### 2.3. ZGE Does Not Influence the Reproductive Capacity of N2

**Figure 3:**
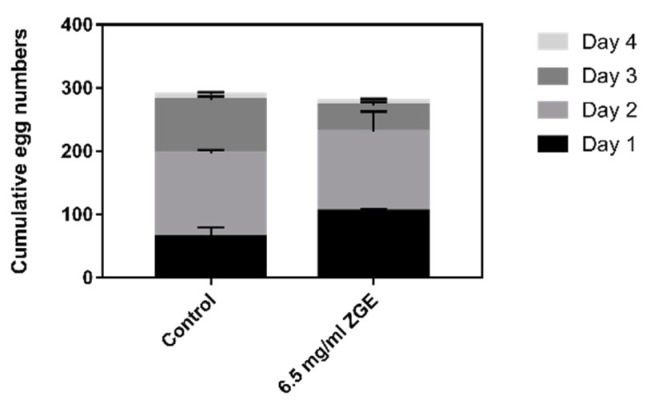
ZGE does not affect the egg laying amount of *C. elegans* in the absence of 5-fluoro-2′-deoxyuridine (FUDR). Worms were fed with *E. coli* and transferred twice a day to prevent overcrowding until the egg laying ceased. The progeny was counted 3 days after the parents were removed, and their numbers were shown as the mean ± SE from three experiments.

Successful reproduction is the ultimate goal of an organism, and therefore the decline in the reproductive capacity of aging is particularly important in the evolution of aging. Reproduction requires germline cell lines and somatic tissue, and thus it is important to understand how the aging of the reproductive system changes and how somatic tissue affects fertility. As shown in Figure 3, for the day of spawning beginning on the first day, the number of offspring in the two groups was not statistically different, and the average ovipositions of the ZGE group and the negative control group were 276.0 and 280.5, respectively. The average spawning of 12 nematodes in the ZGE group was 32 ± 3.78, and the average spawning of the control group was 62.25 ± 15.05 (*p* ≤ 0.01) within the initial 24 h of spawning, which was probably caused by the nutrition provided by ZGE being beneficial to the germ cells of nematodes. However, the number of eggs laid in the last three days was lower than that in the control group. This was attributed to the autologously fertilized androgynous nematodes: about 300 germ cells differentiated into sperm, the remaining germ cells differentiated into oocytes, and the total amount of germ cells of hermaphroditic nematodes was certain. The experimental group and the control group were both found to have a peak growth period of the nematodes on the second day. The comprehensive analysis showed that the supernatant of ZGE had no effect on the reproductive ability of nematodes.

### 2.4. ZGE Improves the Heat-Stress Resistance of C. elegans under Stress Conditions

**Figure 4:**
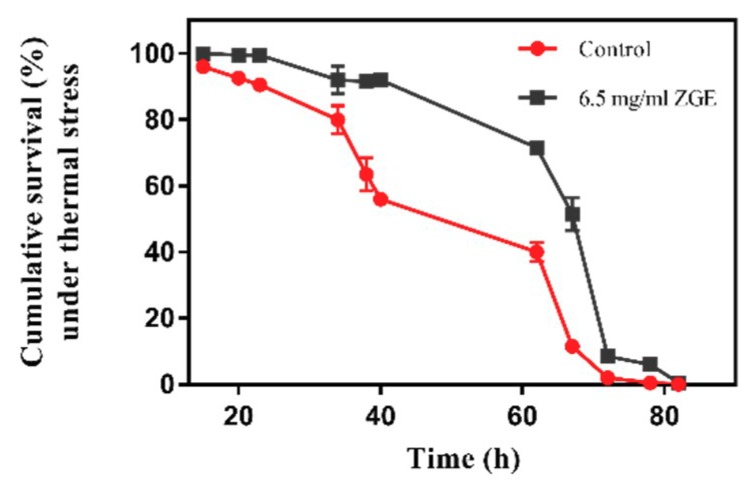
Life span analyses of nematodes exposed to 35 °C in the presence (black) or absence (red) of ZGE.

In order to test whether ZGE can enhance the resistance of nematodes and prolong the survival time of nematodes under high-temperature conditions, the nematodes were kept at 35 °C to simulate the heat stress. As shown in Table 1 and Figure 4, the average survival time of the ZGE group was about 58.89 h and the longest survival time was 82 h. The average survival time of the control group was about 45.32 h and the longest survival time was 77 h. The data showed that ZGE treatment significantly increased the mean survival time of the worms by 29.9%. The results indicated that ZGE could significantly improve the acute heat stress of nematodes. Moreover, ZGE extended the lifespan of nematodes more effectively under the stress environment than normal conditions.

### 2.5. ZGE Enhances the Radiation Resistance of Nematodes

**Figure 5:**
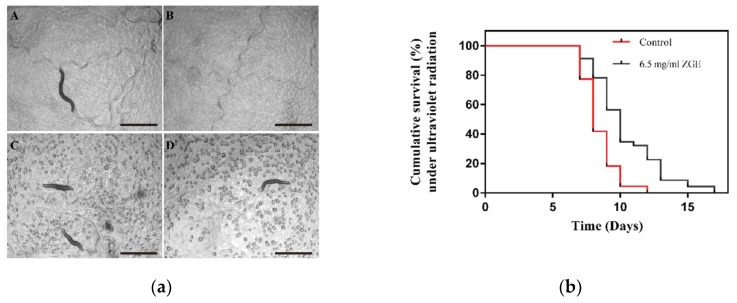
ZGE enhanced the radiation resistance of nematodes. (**a**) Images of physiological states in the ZGE group and the control group on the third day after ultraviolet radiation. (**A**,**B**) represent the ZGE group, and (**C**,**D**) represent the control group. The scale bar was 100 μm. (**b**) Life span analysis of nematodes exposed to ultraviolet radiation in the presence (black) or absence (red) of ZGE. Compared with the control group (*N* = 110), ZGE at a concentration of 6.5 mg/mL (*N* = 115) significantly extended the mean lifespan of wild-type *C. elegans* N2 with a 79.2% increase, *p* < 0.0001 (log-rank test).

In addition to heat shock, a variety of harmful external factors, such as oxidants, heavy metals and ultraviolet radiation, can also cause cellular stress response. Therefore, UV radiation was chosen to stimulate nematodes to figure out whether ZGE could enhance the radiation resistance of nematodes. First, the physiological state of two groups was observed. The body movements of the 6-day-old nematode adults in the ZGE group and the control group showed a coordinated sinusoidal curve, and the pharynx of these two groups was able to shrink in rhythm and aid in eating. The number of contractions was determined to be an average of 290 and 293 times/min. They could sense a series of subtle changes in the environment, such as temperature changes and mechanical stimulation. After the observation, the ZGE group and control group suffered from a dose of 1 kJ/m^2^ of ultraviolet radiation. The pre-experiment showed that if the radiation dose was too low, the nematodes took a long time to die, whereas if the radiation dose was too high, the nematode died too quickly. Therefore, the radiation dose was optimized to be 1 kJ/m^2^. The motor ability analysis indicated that the nematodes after ZGE treatment were able to maintain a rhythmic, active sine curve, and the nematodes’ pharynxes were still able to perform a rhythmic contraction to aid in eating at an average of 256 times/min. However, the nematodes in the control group exhibited slow movement, and the pharyngeal pumping frequency decreased significantly at an average of 162 times/min. On the 9th day (the third day after irradiation), there were significant differences between the two groups in the athletic ability and body posture. As shown in Figure 5a, it was clear that the nematodes were able to carry out rhythmic activity, in which A and B represented the nematodes after ZGE treatment, and the active sinusoidal movement. Meanwhile, the nematodes’ throats were still able to perform a rhythmic contraction to aid in eating. The nematodes in the control group showed physical stiffness and a lack of power, and the number of sinusoidal curves decreased, or even became a straight line. Subsequently, the life cycle showed that 22.7% of the control group died the second day after irradiation and 8.7% died in the ZGE group, while 59% of the control group died the third day after irradiation and 21% died in the ZGE group, as shown in Figure 5b. The longest life cycle of nematodes in the control group was 12 days, and that of the ZGE group was 17 days, while the mean lifecycle of nematodes in the ZGE group was 4.39 days after irradiation, and that of the control group was 2.45 days. The life cycle of nematodes in the ZGE group was up to 79.2% higher than that in the control group. Thus, ZGE could significantly improve the ability of nematodes to resist ultraviolet radiation. Overall, these results indicated that ZGE could enhance the resilience of nematodes and protect nematodes from the damage caused by harsh environments, extending the lifespan of nematodes.

### 2.6. ZGE Can Extend the Lifespan of C. elegans N2 Exposed to High Concentrations of FUDR

**Figure 6:**
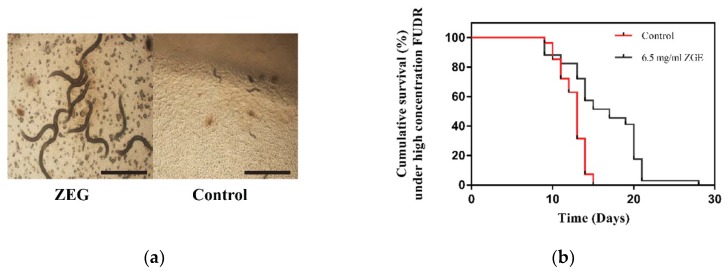
ZGE prolonged the lifespan of wild-type *C. elegans* N2 under a high concentration of FUDR at 20 °C. (**a**) Images of physiological states in the ZGE group (**left**) and the control group (right) exposed to 400 μM FUDR. The scale bar is 500 μm. (**b**) Life span analysis of nematodes exposed to 400 μM FUDR in the presence (black) or absence (red) of ZGE. Compared with the control group (*N* = 54), ZGE at concentration of 6.5 mg/mL (*N* = 68) significantly extended the mean lifespan of wild-type *C. elegans* N2 with a 42.1% increase, *p* < 0.0001 (log-rank test).

FUDR is a commonly used drug to inhibit nematode spawning; the commonly used concentration is 50 μM [37], while a high concentration of FUDR can inhibit the growth of nematode larvae. In order to study whether ZGE has an anti-FUDR effect, we chose 400 μM of FUDR as a damage concentration in the life cycle experiment. As shown in Figure 6a, a high concentration of FUDR could inhibit the normal development of nematode larvae, so that nematodes could not grow normally. In contrast, the nematodes after ZGE treatment were able to grow normally. Figure 6b showed that the longest life cycle of the control group was 14 days, and the maximum life cycle of nematodes in the ZGE group was 28 days. The average lifecycle of nematodes in the ZGE group was 16.47 days, and the average lifespan of nematodes in the control group was 11.59 days. The life cycle of nematodes in the ZGE group was 42.1% higher than that in the control group. Thus, ZGE could significantly improve the ability of nematodes to resist FUDR.

### 2.7. ZGE Promotes the Egg Hatching of C. elegans in the Presence of FUDR

**Figure 7:**
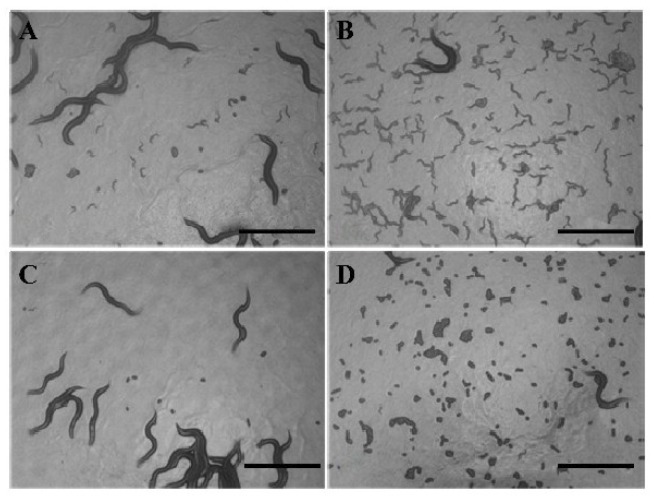
ZGE promotes egg hatching in the presence of FUDR. (**A**,**B**) Images of hatching in the ZGE group, photographed on the sixth day and eighth day, respectively; (**C**,**D**) images of hatching in the control group, photographed on the sixth day and eighth day, respectively. The scale bar is 500 μm.

In order to detect whether ZGE has an effect on FUDR’s inhibition of spawning, 50 μM FUDR was applied to inhibit egg hatching. As shown in Figure 7A,B represent the ZGE group and Figure 7C,D represent the control group. Figure 7A,C were taken on day 6 of the spawning period, and B,D were taken at the end of the spawning period (day 8). The nematodes of the control group could spawn, but the eggs did not hatch. Notably, all the eggs of the ZGE group could hatch. The data indicated that ZGE could resist FUDR and promote the brooding of the nematodes’ germ cells.

### 2.8. ZGE Promotes the Larval Growth of C. elegans in the Presence of FUDR

**Figure 8:**
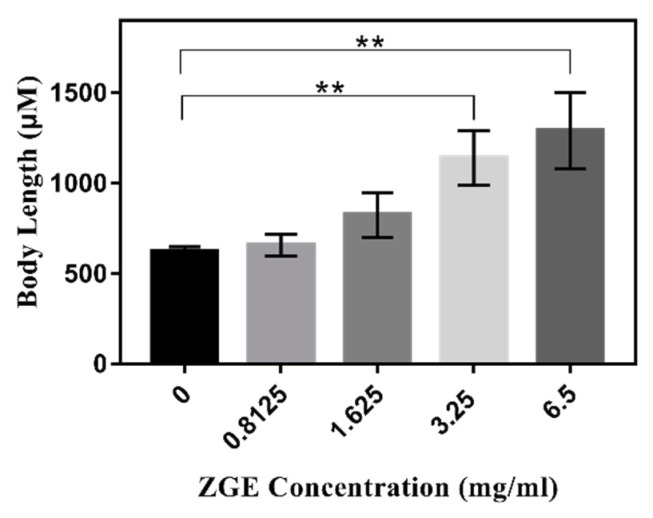
ZGE promoted *C. elegans* larval growth under 100 μM FUDR which inhibited the larvae. ** *p* ≤ 0.01.

In the previous life cycle experiment, with the addition of FUDR, it was found that ZGE could resist the problem of the nematode not developing normally during the larval period, which was caused by the high concentration of FUDR. Therefore, we tried to figure out whether the improvement of ZGE of the growth and development of nematode larvae was conducted in a concentration-dependent manner. In order to verify this idea, the effects of different concentrations of ZGE on the growth of nematode larvae were measured. The results are shown in Table 2 and Figure 8. When the nutrient solution was not added, the average body length of the nematode was about 630 μm, which indicated that FUDR inhibited the normal growth and development of the nematode larvae. When 0.8125 mg/mL of ZGE was used, the average body length of nematodes was about 659 μm, and the effect on growth and development was not obvious. The average body length of the nematode was about 831 μm when the concentration of the nutrient solution of cereal fermentation was doubled to 1.625 mg/mL, which demonstrated that the nutrient solution of cereal fermentation could promote the growth and development of the nematode larvae. Notably, the average body lengths of the nematodes were 1146 and 1284 μm when the concentrations of ZGE were 3.25 and 6.5 mg/mL, respectively. It can be seen from Figure 8 that ZGE promoted the length development of nematode larvae in a concentration-dependent manner.

### 2.9. Total Phenolic Content Analysis

**Figure 9:**
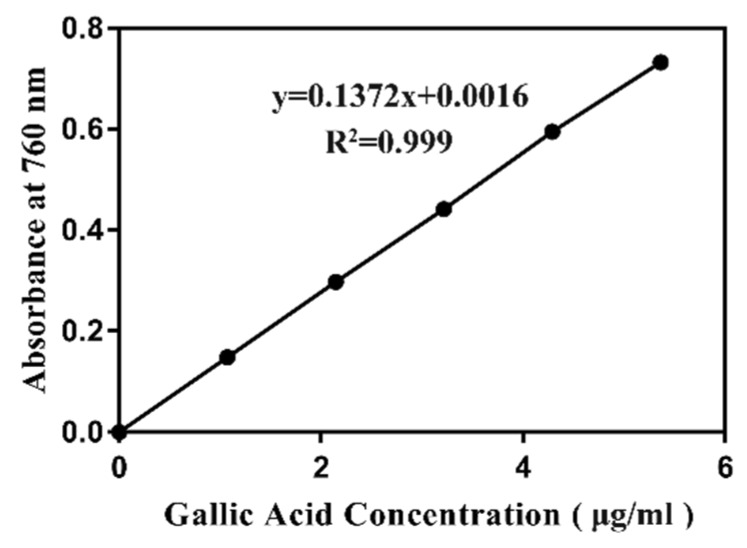
The standard curve of gallic acid based on the absorbance at 765 nm.

A large number of studies have shown a strong positive correlation between polyphenol content and antioxidative ability. To determine whether the antioxidant activity of ZGE was related to polyphenols, we determined the polyphenol content using the Folin–Ciocalteu colorimetric method [38,39]. Gallic acid was used as a standard phenolic compound, and a linear calibration curve of gallic acid with an r^2^ value of 0.999 was constructed, as shown in Figure 9. The polyphenol content in ZGE was expressed as the gallic acid equivalent. The calculated total polyphenol content in ZGE was 28 mg GAE/g (>20 mg/g), which was considered to be a fairly high polyphenol content [40]. However, many natural products with antioxidant activity contained polyphenols at more than 50 GAE mg/g. Thus, the polyphenol content in ZGE was only moderate. From the results, we speculated that the biological activity of ZGE was related to the presence of polyphenols, but other components in ZGE and the interaction between components were also inseparable from the antioxidant activity.

## 3. Discussion

During the past two decades, many reports have shown that a diet with whole-grain cereals plays an important role in preventing the human body from the harm of many chronic diseases including cardiovascular diseases [41], osteoporosis [42], HIV [30], and different kinds of cancers [43]. In addition, cereal consumption has also gained huge appeal for people because of their protective characteristics against the diet and life style associated with disorders such as obesity, diabetes and hypertension [44]. As most of the products are a mixture, the mechanism by which grains exert such evident benefits is not clearly explained, but the main phytochemical components such as polyphenols [45], fibers [46] and vitamin E [47] may exert these essential effects. In this study, we focused on a grain-related product which contains nine different kinds of grains fermented by a mixture of enzymes. In the preliminary work, the analysis of ZGE components by HPLC showed that there were polysaccharides, oligopeptides and other components [30]. Studies have found that the fermentation process could improve the antioxidant properties of plant-based foods, and the main mechanism was that the content of phenolic compounds and flavonoids would increase during the fermentation under the action of microorganisms and enzymes [48]. Simultaneous fermentation has also been applied to increase the phenolic compounds and anthocyanin content in legumes to increase their antioxidant activity [49]. Supernatants and precipitates of ZGE could be obtained separately after centrifugation at 12,000 rpm. According to previous studies of the effects of ZGE on HIV, we found that the composition of the two components was almost identical by detecting and identifying the components of the ZGE supernatant and precipitation by ^1^HNMR spectra, as there were no distinct differences in the ^1^HNMR spectra and the effect on the cell survival in vitro [30]. Therefore, in order to make the aseptic processing of ZGE convenient and facilitate the feeding of nematodes, we selected the supernatant for subsequent nematode experiments. The concentration was determined by extracting 1 mL of the supernatant, drying and weighing it, and the supernatant concentration was 6.5 mg/mL. In this study, we used *C. elegans* as an in vivo model to analyze the anti-aging effect of ZGE, in which the functional effects of ZGE on *C. elegans* did not rely on a single component, and the synergistic interaction between components was the key to function.

It has been proven that the extension of life span in many species, such as yeast, birds, worms and monkeys, is associated with the restriction in caloric intake, a limitation in food and energy intake without malnutrition, at the same time accompanied by a reduction in the risk of diseases associated with human metabolism [50]. If ZGE strongly inhibited the action of *E. coli* OP50, it was likely that its life extension was due to the fact that there was less nematode food on the ZGE-containing plates than on the control plates, thus causing energy limitations. In order to explore whether the changed life cycle of nematodes was caused by the dietary restrictions during the experiments, we examined the effects of ZGE on the growth of food OP50 in nematodes. It was found that ZGE not only did not inhibit the growth of OP50, but also had a slight growth-promoting effect. Thus, ZGE prolonged the life cycle of nematodes, and this was not owing to the energy limitation. If this conclusion were to be further verified, the *eat-2 (ad465)* II mutant would be used, which is usually used as a dietary restriction (DR) model with pharyngeal aspiration defects [51]. If ZGE can prolong the life of the eat-2 (ad465) mutant, we can conclude that ZGE does not function through the DR mechanism.

Next, we tested the effect of ZGE on the lifespan of nematodes under normal conditions. ZGE significantly extended the lifespan in a dose-dependent manner compared with the control group. When nematodes were fed with 6.5 mg/mL ZGE, the average life expectancy of the group fed with ZGE was 23.37 days and the maximum number of survival days was 31 days. However, the average life expectancy and the maximum number of survival days for the control group were only 20.6 and 28 days, respectively. Within 1–12 days, the growth of nematodes in both groups was good. The control group experienced a large number of deaths after 19 days, and more than half of the deaths happened after 20 days. In contrast, a large number of deaths in the ZGE group were present after 24 days, and more than half happened after 25 days. Compared with the control group, feeding ZGE increased the lifespan of nematodes by an average of 2.77 days, an increase of 13.67%. Similarly, when nematodes were fed with 3.25 mg/mL ZGE, the average life expectancy was 24 days, and the maximum number of days of survival was 28 days, which were higher than those values in the control group (the average life expectancy was 22.3 days, and the maximum number of days of survival was 27 days). Feeding ZGE increased the lifespan of nematodes by an average of 1.7 days, an increase of 7.6% compared to the control group. We speculated that ZGE may provide nutrients better absorbed by nematodes or that ZGE can extenuate the senescence caused by the body, causing nematodes to have a better growth status.

In addition to extending life, we also found that ZGE could improve healthspan by determining the effects of ZGE on several physiological indexes during stress, including locomotory capacity, pharyngeal swallow frequency and responses to stresses such as UV-B and thermal stresses. After being pretreated by 6.5 mg/mL ZGE, the nematodes were exposed to UV-B radiation (1 kJ/m^2^) and thermal shock (35 °C). Under these two pressure conditions, the lifespan of the ZGE-treated nematode group was significantly improved compared with the control group, at 79.2% and 29.9%, respectively. In the anti-ultraviolet radiation experiment, we also carried out data statistics on the pharyngeal swallowing of nematodes. On the third day after irradiation, the ZGE group and the control group had significant differences in their motor ability and posture, and it was clear that the nematode could still advance in a rhythmic sinusoid. The pharynx of the nematode was still able to rhythmically contract to aid in eating. In the control group, the nematode was stiff, lacking power and energy. The number of sinusoids decreased or even became a straight line. The above phenomenon clearly indicated that ZGE could prolong the lifespan of nematodes more effectively under the normal environment and maintain the nematodes in a better physiological state.

Further, we conducted another behavior-related experiment to detect the egg production of nematodes under normal conditions and detect egg hatching under pressure conditions caused by FUDR. Many studies have demonstrated that the extension of lifespan was associated with a decrease in fecundity according to the rule of energy saving [52]. However, it should be noted that the nematode stopped the reproduction at its early stage in the life cycle. This suggested that the deterioration of the reproductive system and the loss of reproductive function were probably not caused by the aging of tissues and organs. Huang et al. studied the hermaphrodite mutant nematode and found that the nematode’s autotrophic fertility was not necessarily related to the nematode life cycle, exercise capacity and pharyngeal pumping frequency. Self-fertilization and fertility were determined by the number of genetic germ cells, which were developed between generations, and the utilization of germ cells varied among individuals, suggesting that the process of reproduction was an independent process of development. This was consistent with the experimental results in this experiment, in which the grain-fermented nutrient solution could prolong the life cycle of nematodes without affecting the reproductive capacity of nematodes. Next, we used the chemotherapeutic drug Floxuridine (5′-fluorodeoxyuridine, FUDR) as a kind of stress which was also used to prevent the progeny production at a low concentration (10–50 μM) with a negligible effect on the fourth larval stage nematodes [37,53]. However, when the high concentration of FUDR (400 μM) was given to the nematode, we found that FUDR could inhibit the growth and development of nematodes. Finally, the data showed that, after feeding nematodes with ZGE, the eggs could be hatched under the stimulation of a low concentration of FUDR. Under the stimulation of a high concentration of FUDR, the life span of adults could only be extended by 42.1%, and with the increasing ZGE concentration, the growth of nematode body length was more obvious. Through the above behavioral experiments, we concluded that ZGE could improve the resistance of nematodes, promote the growth and development of nematodes, and improve the physiological state of nematodes under adverse conditions.

Although we have confirmed that ZGE had obvious efficacy in extending the lifespan of nematodes and could significantly improve the resistance of nematodes through a series of experiments, we should further study whether this effect was achieved through antioxidant mechanisms or other aspects. Some key indicators should be detected, such as ROS clearance in nematodes, as well as the regulation of anti-aging-associated genes (*DAF-2, DAF-16, SOD-3, HSP-16.2* and *CAT-1*). It has been reported that the increased expression of *DAF-16* could inhibit insulin/IGF-1-like signaling (IIS) and its receptor *DAF-2* which encodes the insulin receptor family member [54,55], thus increasing stress resistance and longevity. Meanwhile, *DAF-16* played a key role in mediating response against UV-A which could generate ROS [56]. The increased expression of *CAT-1* was associated with the protective effect from stress [57]. As our entire experiment was based on the nematode model, we need to evaluate this model more objectively in future. Although it is widely used and has many advantages, it still has some shortcomings and limitations as a model of human aging. First, owing to the evolutionary difference from humans, some of the genes involved in aging are not homologous to humans [58]. Second, nematodes lack many physiologically important systems, especially the immune system [59]. In general, the nematode is still the best known model for studying aging, and we believe that these restrictions will be overcome in the near future. Moreover, we can also verify the mechanism in parallel with other animal aging models.

In our study, we obtained a grain fermentation solution that acted on *C. elegans,* which was used as a model animal to test its effect on the nematode life cycle. ZGE could not only prolong the lifespan of nematodes under normal culture conditions, but also effectively improved the stress resistance ability of nematodes under stress conditions and improved the physiological state of nematodes. This discovery gives it potential to be used as an anti-aging health product.

## 4. Materials and Methods

### 4.1. Nematode Strains and Maintenance

The wild-type *C. elegans* Bristol N2 and *E. coli* OP50 used in this work were a gift from the Institute of Chemistry, Chinese Academy of Science. *C. elegans* specimens were maintained and assayed (unless otherwise stated) at 20 °C on nematode growth medium (NGM) agar plates seeded with *E. coli* OP50 as a food source. Maintenance and synchronization, as well as egg production assays and 35 °C heat stress tests, were performed in the absence of 5-fluoro-2′-deoxyuridine (FUDR); life span assays (Dillin et al., 2002; Schulz et al., 2007), postembryonic development and adult size assays were performed in the presence of 5-fluoro-2′-deoxyuridine (FUDR). Nematodes (wild-type Bristol N2) were propagated on agar plates containing the respective solvent for four generations before the initiation of experiments. For ZGE supernatant supplementation experiments, the supernatant was added to autoclaved agar at 25 °C as an aqueous stock solution (6.25 mg/mL) to obtain a final concentration of 0–6.25 mg/mL.

### 4.2. Test Drugs and Chemical Reagents

ZGE was obtained from Quanyoujin Biological Technology Co. Ltd. (Tianjin, China). ZGE was produced using white rice, black rice, purple rice, sticky rice, fragrant rice, sorghum rice, Coix seed, millet, and broomcorn millet as raw materials. The steamed and gelatinized grains were saccharified and zymolysed by a mix of glucoamylase (0.25%), lipase (0.05%), protease (0.05%) and amylase (0.02%) at 25–35 °C. When the content of total sugars and total acids was reduced to 16–22 g/mL and 0.38–0.60 g/mL, the fermentation was terminated. The canned ZGE was dispensed to 50 mL into a centrifuge tube by aseptic operation to obtain a ZGE supernatant solution. The ZGE solution was centrifuged at 12,000 rpm for 15 min and sterilized with a 0.22-μm filter. FUDR with a purity of 98% was purchased from Sigma (St Louis, MO, USA). Folin reagent and Na_2_CO_3_ were purchased from Sinopharm Chemical Reagent Co. Ltd. (Shanghai, China) Methanol and gallic acid were purchased from Thermo Fisher Scientific Co. Ltd. (Waltham, MA, USA) and Yuanye Biotechnology Co. Ltd. (Shanghai, China), respectively.

### 4.3. Effects of ZGE on the Growth of OP50 Strain

The OP50 monoclone was inoculated in 10 mL LB liquid medium and incubated at 37 °C overnight. Three conical flasks were prepared containing 20 mL sterilized LB medium, and one bottle was used as a blank control. Two hundred microliters of OP50 and 400 μL of ZGE were added into the second bottle, and 200 μL of OP50 was added into the third bottle, and then cultured at 37 °C at the same time. The value of OD_600_ was detected every 30 min, a total of 5 times.

### 4.4. Life Span Assays under Normal Conditions

All lifespan assays were conducted at 20 °C on solid NGM and were replicated in at least three independent experiments. The worms in the treatment groups were grown on NGM plates coated with ZGE (3.25 and 6.5 mg/mL) diluted in a suspension of live *E. coli* OP50. The worms in the control group were grown on NGM plates coated only with OP50. To obtain a synchronous population, pregnant nematodes were placed on NGM plates with *E. coli* OP50 on the surface and allowed to lay eggs for approximately 3 h at 20 °C [60]. One hundred eggs per plate were transferred to two freshly seeded *E. coli* OP50 plates, and then the eggs were kept at 20 °C for 48 h to develop into L4 larvae. Then, 12–15 healthy L4 larvae worms were transferred to a freshly seeded treatment plate immediately before they entered the spawning period. The worms were kept at 20 °C, which was marked as the first day. We screened and transferred parents every other day for the first 10 days. *C. elegans* individuals were considered to be dead when they did not respond to prodding with the tip of a bristle. Animals that crawled off the plate, exploded, bagged, or became contaminated were censored.

### 4.5. Self-Brood Size and Rate of Egg Production under Normal Conditions

We picked 12 L4 stage nematodes from the ZGE group and control group, respectively, for the corresponding plates. Then, we placed one nematode per plate, which was numbered and recorded at a constant temperature incubator at 20 °C. We recorded the day of spawning as the first day. The nematode was transferred to the new corresponding plate daily until the end of the spawning period of the nematode, and this process lasted 4 days approximately. We hatched all plates at 20 °C and counted the total number of nematodes hatched by each nematode in 4 plates; that is, the number of eggs laid by the nematode.

### 4.6. Heat-Shock Assays

To evaluate the potential longevity-extending effect of ZGE on wild-type *C. elegans* N2 under high-temperature conditions, heat-shock assays were performed using 2-day-old adult worms at 35 °C, which was considered to be a heat stressor [61]. The worms that just reached adulthood were pretreated with 6.5 mg/mL ZGE for 48 h and then transferred to an incubator at 35 °C. For all the lifespan assays, each experiment was repeated three times and conducted in a double-blind manner.

### 4.7. Anti-Ultraviolet Radiation Assays

The synchronized nematodes were placed on the ZGE group and the control group culture plate at 20 °C, respectively. On the 6th day of culture, the dose was 1 kJ/m^2^ UV irradiation. The nematodes were observed by Olympus IX73P1F fluorescence microscopy, and the number of nematodes killed was recorded.

### 4.8. Effects of ZGE on the Life Cycle of Nematode with High Concentration FUDR

The synchronized eggs were placed in the ZGE group and the control group NGM plate, and cultured at 20 °C with 400 μM FUDR in these two groups. Life cycle observation was conducted according to the method in Section 4.4.

### 4.9. Effects of ZGE on the FUDR Influence on Nematode Egg Hatching

The synchronized eggs were placed in the ZGE group and the control group NGM plate and cultured at 20 °C. The two groups both contained 50 μM FUDR and were recorded as day 0. The group setting and statistical method are explained in Section 4.5.

### 4.10. Effects of ZGE on the Growth and Development of Nematodes Influenced by FUDR

The synchronized eggs were placed on the NGM plates with ZGE supernatant concentrations of 0, 0.8125, 1.625, 3.25 and 6.5 mg/mL, and cultured at 20 °C with 100 μM FUDR in each group. When the body length of the nemotodes was no longer growing, each group of nematodes was picked up to add to the glass slides containing 0.1% sodium azide. The nematode was straightened with the tip of the gun. The nematodes were observed by Olympus IX73P1F fluorescence microscopy and the length of each group was calculated.

### 4.11. Folin–Ciocalteu Method for the Determination of Total Polyphenols

The test sample and standard samples were prepared separately. For the test solution, an appropriate amount of uniformly mixed sample was weighed in a centrifuge tube and 5 mL of 70% methanol aqueous solution was added and preheated at 70 °C. Then, the mixture was stirred well with a glass rod, immediately transferred to a 70 °C water bath, and dipped for 10 min (each stirred once every 5 min), removed and cooled to room temperature, centrifuged at 4000 r/min for 10 min, and the supernatant transferred to a 10 mL volumetric flask. The residue was re-extracted with 5 mL of 70% aqueous methanol solution, and the combined extracts were made up to 10 mL, shaken, and passed through a 0.45 μm filter to be tested. For the standard preparation,1 mL, 2 mL, 3 mL, 4 mL, 5 mL of 1000 mg/L gallic acid standard stock solution were accurately transferred into a 100 mL volumetric flask, and diluted to volume with water, well shaken, making final concentrations of 10, 20, 30, 40, and 50 μg/mL, respectively. One milliliter of gallic acid working solution was pipetted with water or test solution into a graduated test tube, and 5 mL of forinol (10%) was added to each test tube and well shaken. Within 3–8 min, 4 mL of 75% Na_2_CO_3_ solution was added, water added to the mark, and the mixture was shaken. After standing for 60 min at room temperature, the absorbance was measured with a spectrophotometer (UV-vis spectrophotometer, Agilent 8453, Foster City, CA, USA) at a wavelength of 760 nm using a 10 mm cuvette. A standard curve was prepared based on the absorbance of the gallic acid working solution and the concentration of each working solution. Next, we calculated the total polyphenol content in the sample according to the formula below:

$$X=\frac{\left(A-A_{0}\right)\times V\times d\times 100}{k\times m\times 10^{6}}$$

where X is the content of total polyphenols in the sample, g/100 g; A is the absorbance of the sample extract; A_0_ is the absorbance of the blank; k is the slope of the gallic acid marker; d is the dilution factor. M is the the amount of the sample, is g; and V is the sample extract volume, mL.

### 4.12. Statistical Analysis

The data obtained from the above experiments were collected three times or more. The experimental data were analyzed and plotted using Excel and GraphPad Prism 7.0 software. The Kaplan–Meier analysis was performed on one of the datasets from the life cycle experiment; the significance test was performed using the t test. * *p* ≤ 0.05, ** *p* ≤ 0.01, *** *p* ≤ 0.001 were considered to be statistically significant.

## 5. Conclusions

In this paper, we proposed, for the first time, anti-aging properties and nutrient activity of nutraceutical ZGE, which was obtained by fermenting nine grains. In this study, we identified that ZGE could prolong the life span of nematodes under a normal or damaging environment at high concentrations of FUDR. Meanwhile, ZGE could also promote nematode germ cell hatching in the presence of 50 μM FUDR. Besides this, the resistance to heat shock and radiation of nematodes was enhanced after the ZGE treatment. All of these interesting findings suggested that ZGE could improve the cellular defense of nematodes and that the polyphenols in ZGE may exert a potential effect. Therefore, ZGE is a special food that can not only provide nutrition for human but also has antioxidant potential and prospective use in the human body. The related molecular mechanisms will be further studied in subsequent experiments.

## 6. Patents

This research was authorized by CNIPA (Grant No. ZL 2014 1 0408471.6).

## Figures and Tables

**Table 1:** ZGE’s effects on the survival rate of adult *C. elegans* under thermal stress. *** *p* ≤ 0.001.

ZGE Treatment	Thermotolerance, 35 °C (h)		
(mg/mL)	Mean	Maximum	Mean Fold Increase/%
0	45.32 ± 18.2 (46)	77	--
6.5	58.89 ± 14.24 (61)	82	29.9 ***

**Table 2:** ZGE promoted *C. elegans* larval growth under conditions in which FUDR inhibited the larval growth.

Group (mg/mL)	0	0.8125	1.625	3.25	6.5
Body length (μM)	630 ± 24.05 (*n* = 27)	659 ± 86.27 (*n* = 30)	831.1 ± 124.7 (*n* = 27)	1146.11 ± 154.78 (*n* = 33)	1284.74 ± 215.18 (*n* = 35)

## Abbreviations

ZGE	Zymolytic grain extract
FUDR	5-fluoro-2′-deoxyuridine
ROS	Reactive oxygen species
*C. elegans*	*Caenorhabditis elegans*
